# The Uterus

**Published:** 1858-11

**Authors:** Tyler Smith


*The Uterus.* By Tyler Smith.

The uterus is in relation with the cerebral, spinal, and ganglionic divisions of the nervous system, and posesses properties derived from each of these sources of motor power.

In the first place let us consider the relation of the cerebral system to uterine motor action, as seen in the influence of volition and emotion.

The uterus is withdrawn from the direct influence of volition. The will has no direct power either to contract or to dilate this organ. Labour may take place when cerebral paralysis exists, the will being entirely in abeyance, but the uterine movements depend on reflex action and peristaltic action remaining perfect. But, though not exerting any direct influence, volition may affect the uterus indirectly. In certain cases of uterine inertia, when the contractions of the uterus have entirely ceased, voluntary efforts are sometimes sufficient to reproduce uterine contractions. Efforts at expiration, with the glottis closed, cause the abdominal muscles to compress the uterus mechanically, and this compression stimulates the uterus in the same way as manual irritation of the organ. What is called in other organs, consensual action, may also probably be excited in the uterus, to some degree by volition. Violent voluntary action quickens the action of the heart, and the voluntary contraction of the internal rectus muscle contracts the iris, though both the heart and the iris are removed from the direct action of volition. In a similar manner, the uterus, during parturition, is probably affected by the intense efforts at expiration and bearing down, which accompany the pains of labour.

A very powerful influence may be exerted upon the uterus by emotion. A fright or any violent mental disturbance, may bring on labour prematurely, or produce abortion. During labour, any sudden emotion of the mind may increase or arest uterine action. The different effects of hope or despair on the commencement, progress and termination of labour, have frequently been remarked. Emotion often plays the part of Tantalus to the accoucheur. His entrance into the lying-in room may arrest the pains of labour for a time, through the influence of emotion ; but if he should leave the house, they as often return with increased vigor, and terminate the labour abruptly in his absence. After delivery, the maternal emotion exerted by the sight of the infant, causes the uterus to contract in a remarkable manner. Emotional, like voluntary action, is psychical in its nature, and originates in the cerebrum ; but it acts upon the uterus and other parts through the spinal marrow, the great organ of physical motion. This is evident from the fact that emotional movements may occur in parts whieh are entirely paralyzed to cerebral voluntary motion.

Let us now refer to the forms of uterine action depending upon the the spinal marrow, a subject which did not admit of comprehension before the beautiful discovery of the spinal or disastatic system by Dr. Marshall Hall.

The reflex spinal or diastalic action of the uterus is excited in various modes; and it is upon this form of contraction, aided by peristaltic action, and the extra-uterine reflex actions excited during the process, that natural parturition essentially depends. Contraction of the uterus of a reflex or diastalic kind, may be excited by irritation of the mammæ, as in the act of suckling the infant; by the impression of cold upon the vulva or abdominal surface; by irritation of the rectum, as by a stimulating enema; by gastric irritation, as in drinking a gulp of cold water, or swallowing a piece of ice; by ovarian excitement, as in the occurrence of abortion from the menstrual nidus; by irritation of the vagina or pressure on the perineum; and by irritation of the os and cervix uteri. These facts supply the proof that the uterus is in relation with mammary, pubic, rectal, pneumo-gastric, ovarian, and vaginal nerves, and the nerves of the os and cervix uteri, as incident excitor nerves. There can be no doubt that in an organ thus subject to reflex action, its own nerves are excitors, and that in all contractions of the uterus excited by irritation of the internal surface of the uterus or of the os and cervix during the passage of the fœtus, the uterine actions are both reflex and peristaltic. That the internal surface of the uterus possesses incident spinal nerves, is proved by the occurrence of vomiting, etc., from uterine irritation. There is indeed no instance of a mucous surface wanting the power of exciting reflex action in other parts of the body. It is a question if any pure spinal fibres reach or proceed from the uterus, unmixed with fibres from the ganglionic. This admixture produces a curious effect upon the reflex contractions of the organ. If we irritate the conjunctiva with a feather, the orbicularis muscle contracts instantly. If we tickle the fauces, efforts at vomiting are immediately produced. But in the case of the uterus, contraction does not follow upon the irritation, in so sudden a manner. I have sometimes in cases of alarming hemorrhage, had my hand in the uterus for a considerable time, and have carefully watched the influence of reflex stimuli upon the uterus. If, while the uterus remains flaccid, cold water is sprinkled upon the face, the uterus does not act at once, but after an interval of half a minute to a minute, or even longer, the organ slowly begins to contract, reaches its acme by degrees, and as slowly relaxes. The same thing happens if, while the hand remains *in utero*, cold or iced water be injected into the cavity of the organ.

As a motor organ, the uterus stands alone in many respects. Unlike the rectum and bladder, it is not directly influenced by volition; and, unlike the heart, it is extremely prone to reflex action. It more nearly resembles the œsophagus, which is uninfluenced by the will, but is endowed with reflex motion and peristaltic action. It differs, however, from the œsophagus in the number of excitor surfaces with which the spinal system places it in relation. There is no other organ—not even the stomach—which can be excited by so many distinct organs, or which acts as such an extensive excitor of motor action in other parts, both in the impregnated and unimpregnated states, as the uterus.

Besides the reflex action of the spinal marrow, and its system of excitor and motor nerves, there is the direct action of the spinal centre to be considered, though this form of spinal action does not play the important part assigned to it by Serres, Brachet, and Segalas. In what is termed direct or centric spinal action, the spinal centre with its motor nerves are concerned, to the exclusion of the incident or excitor nerves. Various instances of centric spinal action may be given. Thus, ergotine passing into the blood, affects the spinal centre, and its effects reach the uterus by its motor nerves. Other oxytoxic agents such as strychnia, carbonic acid, savin, aloes, alcohol, the biborate of soda, and probably ipecacuanha, act in a similar manner. The state of the circulation affects the spinal centre in a very distinct manner. It is well known that there is one form of puerperal convulsion depending upon hæmorrhage, where the heart and great vessels have been nearly emptied of blood, and another caused by fulness of circulation. The convulsion probably depends greatly upon the influence of deficiency or excess of blood in the vessels of the nervous centres. Want or excess of blood, or materies morbi in the circulation, act, then, as direct stimuli to the spinal centre, and in this way the state of the circulation affects the uterus during labor. The uterus acts with increased force when the circulation is either plethoric or anæmic; though in the latter case, exhaustion of its nervous energy quickly ensues.

We now come to the consideration of the peristaltic or ganglionic motor actions of the uterus.

When any part of a muscular organ, supplied in whole or in part by the ganglionic system of nerves, is irritated, the contraction which ensues generally spreads in a vermicular manner to a distance from the point of irritation, and continues for some time after the exciting cause is removed. This is called peristaltic motion or action. The uterus is eminently endowed with this peristaltic form of contraction. When one point of the uterus is stimulated, through the abdominal parietes, or by the introduction of the hand into the uterus, the contraction excited extends to the whole organ. Harvey described this peristaltic action of the uterus in the doe. William Hunter saw it in the cat and the rabbit. Muller observed it in the uterus of the rat and the oviduct of the turtle; and I have seen it in the uterus of the guinea-pig and other animals. The heart, œsophagus, and intestine may be excited to contraction after death; and I have seen the uterus and vagina of the rabbit contract rhythmically, when irritated, several hours after the cessation of respiration. Many cases are on record in which women have died undelivered, but the child has been expelled spontaneously after death. In one case a woman dying during labour was placed in a coffin, and the fœtus was found the next day perfectly expelled. This *post mortem* parturition most generally depends either upon peristaltic action commencing after the occurence of somatic death, or upon the rigor mortis affecting the uterus. It is well known that the rigor mortis affects the other involuntary muscles, and especially the heart, which is contracted by this influence to such an extent as to empty the ventricles, and even to stimulate concentric hypertrophy Cases are related in which the fœtus has apparently been expelled some days after the death of the mother by the gaseous distension of the abdomen; but these are different from cases occuring shortly after death, and before decomposition has set in. In the living subject, the peristaltic action of the uterus is the basis of the other uterine actions. In natural labour it is combined with reflex uterine action, and with various forms of extra-uterine action; but, under certain circumstances, it appears able to effect the expulsion of the child without other aid. In paraplegia from disease of the lower part of the spinal marrow, or in animals reduced to the same state by experiment, the peristaltic action is the chief power remaining to the uterus. In such case, delivery has been effected in an imperfect manner by the peristaltic action of the uterus, or the application of galvanism to the organ. It is not, however, known how much of the spinal marrow must be destroyed before the reflex or diastaltic actions of the uterus cease.

[We have made the above selection, not only on account of the intrinsic value of the explanations given of uterine action, but, because of its bearing upon the interesting case of fœtal expulsion, reported in the first No. of this Journal, by our esteemed collaborator Dr. B. F.

Fessenden. It will be seen that "many cases are on record in which *post mortem* parturition has taken place," and that the phenomenon can be easily explained on philosophical principles.—*Editor.*]—*N. C. Med. & Sur. Journal.*

